# Metric-based vs peer-reviewed evaluation of a research output: Lesson learnt from UK’s national research assessment exercise

**DOI:** 10.1371/journal.pone.0179722

**Published:** 2017-07-11

**Authors:** Kushwanth Koya, Gobinda Chowdhury

**Affiliations:** iSchool @ Department of Computers and Information Sciences, Faculty of Engineering and Environment, Pandon Building, Camden Street, Northumbria University, Newcastle City Campus, Newcastle-upon-Tyne, United Kingdom; Universidad de las Palmas de Gran Canaria, SPAIN

## Abstract

**Purpose:**

There is a general inquisition regarding the monetary value of a research output, as a substantial amount of funding in modern academia is essentially awarded to good research presented in the form of journal articles, conferences papers, performances, compositions, exhibitions, books and book chapters etc., which, eventually leads to another question if the value varies across different disciplines. Answers to these questions will not only assist academics and researchers, but will also help higher education institutions (HEIs) make informed decisions in their administrative and research policies.

**Design and methodology:**

To examine both the questions, we applied the United Kingdom’s recently concluded national research assessment exercise known as the Research Excellence Framework (REF) 2014 as a case study. All the data for this study is sourced from the openly available publications which arose from the digital repositories of REF’s results and HEFCE’s funding allocations.

**Findings:**

A world leading output earns between £7504 and £14,639 per year within the REF cycle, whereas an internationally excellent output earns between £1876 and £3659, varying according to their area of research. Secondly, an investigation into the impact rating of 25315 journal articles submitted in five areas of research by UK HEIs and their awarded funding revealed a linear relationship between the percentage of quartile-one journal publications and percentage of 4* outputs in Clinical Medicine, Physics and Psychology/Psychiatry/Neuroscience UoAs, and no relationship was found in the Classics and Anthropology/Development Studies UoAs, due to the fact that most publications in the latter two disciplines are not journal articles.

**Practical implications:**

The findings provide an indication of the monetary value of a research output, from the perspectives of government funding for research, and also what makes a good output, i.e. whether a relationship exists between good quality output and the source of its publication. The findings may also influence future REF submission strategies in HEIs and ascertain that the impact rating of the journals is not necessarily a reflection of the quality of research in every discipline, and this may have a significant influence on the future of scholarly communications in general.

**Originality:**

According to the author’s knowledge, this is the first time an investigation has estimated the monetary value of a good research output.

## Introduction

Research is evaluated in several forms and despite years of debate to find an effective and efficient method, the academic community is yet to reach a consensus. Peer review has been the oldest form of research evaluation and stands firm in spite of several disputes surrounding its functioning. Several databases and metrics such as Web of Science, Scopus, Scholar, InCites, SciVal, h-index and Altmetrics attempt to establish the quality of research through publication profile and citation profile or both [1–3]. However, these measures remain questionable due to the narrow interpretations they produce and are often confined to academic evaluation [4–6]. Consequently, some alternative approaches such as the Web-impact metrics, societal impact, and a combination of principles such as the Leiden Manifesto were proposed [1, 2, 7]. Funding good research is essential for the survival of science, and progressive countries, invest between 2% to 3% of their gross domestic product on research and development activities, a good portion concentrated in Higher Educations Institutions (HEIs), which has proven to be extremely beneficial for multiple societal aspects [8]. However, two fundamental questions still remain unanswered, viz. (1) what is the economic value of a research output, as perceived by governments or agencies that fund research, and (2) what makes a good research output, and more specifically is there a direct relationship between the quality of a research output, as determined through its monetary value, and the source of its publication This study aims to address the following questions:

1. What is the monetary value of an output showcasing good research?
2. Does the value vary amongst different disciplines?
3. Is there a relationship between the value of a research output and the reputation of its publication source; and
4. Can the assigned value of a research output alter the nature of science and research in a country?

The recently concluded national research evaluation exercise in UK, called REF2014, has been used as a case study to find answers to these questions.

### UK universities

Like in any other countries, HEIs play an essential role in UK society. According to a latest Universities UK report, the UK HE sector contributed £39.9 billion, equivalent to 2.8% of the UK’s gross domestic product (GDP), and employed 757, 268 individuals in 2011 [9]. According to the Higher Education Statistics Agency (HESA), a typical UK HEI’s revenue break-down is as follows: 35% tuition fee, 30% funding council grants, 16% research grants and contracts, 1% from endowments/ investment income, and 18% from other sources i.e. alumni donations etc. [10]. Visibly, a large portion of revenue for the HEIs come from the funding councils, which generally award the funding based on performance, thus making research evaluation and the financial returns of research conducted an important question for academia to inquire. Considering the recently concluded Research Excellence Framework (REF) 2014, the UK’s national research assessment exercise, as a case study offers a chance to answer the question and also an opportunity for other research intensive countries to compare their performance-based research funding. It may be argued that the amount of money available to distribute to the HEIs is very much dependent on the available budget for a particular government, and hence the monetary value of the research outputs will not provide us a definitive figure, and therefore may not be applicable to others. However, since the REF 2014 is a national exercise, and it determines the annual funding for research for all the HEIs, and all the HEI staff in the country, for six to seven years (until a similar exercise, or an alternative, takes place), it has an impact on the research and scholarly activities of the entire country for several years. Hence, we decided to use the REF2014 datasets to find answers to the research questions mentioned earlier.

### What is the REF 2014?

The REF 2014 was a research evaluation exercise conducted by a combined team of organisations, namely the Higher Education Funding Council for England (HEFCE) and Wales (HEFCW), the Scottish Funding Council (SFC) and the Department for Employment and Learning (DEL) of Northern Ireland to measure the quality of research at various HEIs in the United Kingdom [11]. It is a performance-based HEI research funding system whose results inform the higher education funding bodies to allocate funding each year to HEIs based on their performance [7]. It also plays a vital role in an HEI’s ability to secure funding from other sources, league table scores, reputation and attracting talent in terms of students and academics [12].

The results from the current REF assisted in the yearly disbursal of £1.6 billion per year to UK based higher education and research institutions until the next such exercise, possibly commissioned for 2021. The results of REF 2014 led to drastic alterations in funding allocations when compared to the previous REF (RAE 2008). An HEI lost about 17.1% (£14.2 million) of its funding and in another exceptional case, an HEI lost 45% of its funding. The maximum gain by any HEI stood at 12.4% (£7.1 million) [13, 14]. The repercussions of such fluctuations are considerable to the future of research at UK HEIs.

To submit for the REF, the HEIs had to choose the areas of research (called Units of Assessment/ UoAs) out of the available 36 UoAs, which they wished to be evaluated upon and prepared their submission in a prescribed format. The submissions for the REF 2014 were evaluated by 1052 individuals, of which 77% were academics and 23% were users (individuals who apply HEI research and collaborators outside academia), under the guidance of 36 expert sub-panel chairs, additionally supported by four main panel chairs to evaluate and determine the quality of research. Research was adjudged into five categories; 4* (world leading), 3* (internationally excellent), 2* (recognised internationally), 1* (recognised nationally) and unclassified (REF, 2014). The overall quality of research was assessed through a combination of quality of research outputs (65% weightage) in terms of rigour, originality and significance; ‘impact’ of research (20% weightage), a new factor introduced in REF evaluation, assessing the ‘reach and significance’ of research on multiple societal factors; and research environment (15% weightage), in terms of ‘vitality and sustainability’ i.e. PhD completions, laboratory facilities and wider disciplinary contributions [11].

### Research outputs

HEIs submitted various types of research outputs for evaluation i.e. journal and conference articles, books, book chapters, edited books, patents, design, artefacts, software, exhibitions and compositions etc. The submitted outputs were evaluated and graded into five categories as previously mentioned. However, only 4* (world leading) and 3* (internationally excellent) outputs were eligible for funding and the final weightage, fairly taking into account the number of staff members who had submitted for the UoA from the HEI, thus minimising quantitative bias. Finally, funding is allocated based on the weightage acquired by the HEIs, which is a sum of the number of 3* outputs and four times the number of 4* outputs. According to HEFCE’s pre-submission guidelines, 4* outputs received four times higher funding than 3* outputs and the allocation of funding varied across disciplines as research expenses vary in different research disciplines (for example, laboratory-based research incurs higher expenses than library-based research). Post-REF 2014 data reveals that research outputs alone led to a total allocation of £661.3 million Pounds in research money to UK HEIs per year, not considering the ‘London weighting’ which was exclusively granted to HEIs located in London due to higher costs associated with the capital.

### Evaluation of quality of the output

According to HEFCE, the outputs were evaluated upon their ‘originality, significance and rigour’ in comparison to international standards [11, 15]. HEFCE advised HEIs against choosing outputs with high citation indices for submission, rather select outputs which the HEIs affirm as high quality. However, in some cases, the citation data of outputs and significance of outputs beyond academia were considered as indicators of quality by the sub-panels [15].

## Methods

Six HEIs were randomly chosen from each of the 36 UoAs of the REF. The HEIs’ percentage of 4* (X) and 3* (Y) research outputs was noted from the REF’s results, in addition to considering the staff count (A) of each HEI. This allowed the calculation of the number of 4* (B) and 3* (C) outputs considered for weightage (W).

$$B=\left(\frac{X}{100}\right)*A;\;C=\left(\frac{Y}{100}\right)*A$$

Weightage (W) was calculated as the sum of four times the number of 4* and 3* research outputs.

W=(B*4)+C

The total funding awarded (FA) for each HEI under each UoA was noted from HEFCE’s funding allocation table (http://www.hefce.ac.uk/funding/annallocns/1516/research/). Assuming all outputs to be rated 4*, the value of each 4* output (F) is obtained by dividing the total funding award (FA) by the weightage (W).

F=FA/W

According to the HEFCE, the value of each 4* output is four times the value of each 3* output. Hence, the value of each 3* output (T) is obtained by dividing the value of each 4* output (F) by 4.

T=F/4

### Example of the calculation

For clarity of the above calculation, let us consider the case of the University of Cambridge under the General Engineering UoA. 37.4% (X) of its research was rated 4* and 55.8% (Y) was rated 3* with a staff count of 177.20 (A) FTE. It received £5,328,295 (FA) from HEFCE for its outputs performance in General Engineering. The number of 4* (B) and 3* (C) outputs considered for weightage (W) can be obtained as follows

$$B=\left(\frac{37.4}{100}\right)*177.2=66.27;\;C=\left(\frac{55.8}{100}\right)*177.2=98.88$$

Thus weightage is obtained by the sum of four times the number of 4* and the number of 3* outputs.

W=(4*66.27)+98.88=363.97

Assuming all outputs were awarded a 4* rating, the value of each 4* output (F) is obtained by dividing the total funding received (FA) by the weightage (W). As 4* outputs are four times the value of 3* outputs, the value of each 3* (T) output is obtained by dividing the value of each 4* output by 4.

$$F=\frac{£5328295}{363.97}=£14639.38;\;T=\frac{£14639.38}{4}=£3659.84$$

At this point, it is important to understand REF’s instructions. The REF required each staff member considered for submission to submit four outputs each [15]. Cambridge for the General Engineering UoA presented 177.20 FTE staff and submitted 616 outputs. Ideally, Cambridge should have submitted 708.8 (No. of staff submitted multiplied by 4). However due to specific circumstances (i.e. career breaks, early career researchers etc.) submitted 616 outputs for evaluation. The REF is aware of such circumstances and is considerate by not penalising the HEI, taking into account the phase of a researcher’s career and personal circumstances. The REF further calculates the rating based on the ideal number of submissions, but not the actual number of submissions. In the case of Cambridge, 37.4% of the ideal 708.8 were rated 4* and 55.8% of the ideal 708.8 were rated 3*, which takes the number of 4* submissions to 265.09 and the number of 3* submissions to 395.51.

So, the total funding for outputs in the General Engineering UoA for Cambridge was

(265.09*1439.43)+(395*3659.86)=£5328295

By multiplying the number of 4* and 3* submissions with their respective value and adding them up will give us the final amount of funding acquired by Cambridge. In other words, if every member of Cambridge staff had to submit 4 outputs they would get the same amount of money that they have received with lower number of output (616 as opposed to 709) because of specific staff circumstances. This not only is a simplified explanation of the REF’s working, it also corroborates our calculations about the value of each 4* and 3* output.

### Design to observe relationship between funding awarded and publication source of the outputs

As it was impossible to identify the REF rating of an individual output, we performed an indirect measure by investigating the proportion of HEIs submitted outputs published in quartile-one (Q1) journals and its relationship to funding acquired. We decided to find out whether any direct relation existed between the monetary value of an output and the reputation of its source of publication as measured through journal impact factor.

Five REF UoAs; clinical medicine (Panel A, UoA 1), physics (Panel B, UoA 9), psychology/psychiatry/neuroscience (Panel A, UoA 4), anthropology/development studies (Panel C, UoA 24) and classics (Panel D, UoA 31) were chosen from the available 36 UoAs under four main panels. Each chosen UoA came under each main panel of assessment, except clinical medicine and psychology/psychiatry/neuroscience which came under panel A.

However, since it was not possible to get the necessary data directly from the REF2014 results, i.e. it was not possible to find out which output got a 4* rating, we decided to use an alternative approach. By using the Thompson Reuter’s Journal Citation Report against the submitted journal papers for each HEI in the chosen UoA, we identified how many of the submitted articles were in top quartile journals, and accordingly we prepared a rank list of the HEIs in a given UoA based on the number of Q1 publications. This list was plotted against their percentage of 4* outputs to find any relationship.

All the journal articles submitted by English HEIs in each of the UoA—10986 for Clinical Medicine; 5302 for Physics; 7484 for Psychology, Psychiatry and Neuroscience; 1198 for Anthropology and Development Studies; and 345 for Classics—a total of 25315 articles and their corresponding journal’s quartile score was noted through Thomson Reuter’s Journal Citation Reports. Quartile score is calculated for each journal in every subject category according to the quarter where its impact factor falls under. Thus, a quartile 1 (Q1) journal is one whose impact factor falls in the top 25% of all journals within the same subject category. The quartile scores of all the journals for the year 2013 were considered for this study as the quartile scores for 2014 came out in mid-2015 and the only data available for sub-panel members during the REF evaluation in 2014 would have been the data from 2013. Some journals cannot completely associate with a single specific subject category. In such cases the nearest related subject category to the UoA was considered while noting the quartile scores.

Subsequently, all the journal articles submitted by the HEIs, whose journals were in the Q1 category were considered, allowing the calculation of all the HEIs percentage of Q1 publications, which was compared against percentage of 4* publications.

### Data

All the data for this study is sourced from the openly available publications which arose from the digital repositories of REF’s results and HEFCE’s funding allocations.

### Statistics and analysis

All the data for the value calculation part of the study were transferred from sources and analysed using the formulas feature in MS Excel. For the next part of the study MS Excel assisted in transferring the data from sources and calculation of the HEIs percentage of Q1 publications. Thereafter, the data was visualised using IBM’s SPSS Statistics 22, in addition to verifying the linear relationship between percentage of Q1 publications and funding awarded per FTE staff in HEIs using a bivariate Pearson correlation test [16, 17].

## Results & discussion

### Monetary values of good research outputs in various UoAs

Using the formula mentioned in the previous section, it was noted that each internationally excellent output (3* in the parlance of REF2014) was awarded between £1876 and £3659, whereas a world leading output (4* in the parlance of REF2014) was awarded four times the award for an internationally excellent output, between £7504 and £14639, varying according to the UoA (discipline). Engineering based subjects, pure and environmental sciences were the highest earners; £3659 for internationally excellent and £14639 for world leading outputs. Humanities, language and area studies were the lowest earners; £1879 for internationally excellent and £7504 for world leading outputs. Health related subjects, clinical medicine, biological and agricultural sciences received £3280 and £13123, for an internationally excellent and world leading output respectively. The financial awards for outputs in the remaining subject areas are described in Table 1. The awards for the outputs presented are for a one year period. For an entire assessment period, the outputs will fetch six times the figures stated above, realistically assuming an assessment period to be six years in the UK.

**Table 1:** Value of each 3* and 4* output in different units of assessment.

Units of Assessment (UoA)	Internationally excellent £	World leading ££££
27. Area Studies 28. Modern Languages and Linguistics 29. English Language and Literature 30. History 31. Classics 32. Philosophy 33.Theology and Religious Studies 36. Communication, Cultural and media Studies/Library and Information management	1876.19–1876.55	7504.78–7506.22
18. Economics and Econometrics 19. Business and Management Studies 20. Law 21. Politics and International Studies 22. Social Work and Social Policy 23. Sociology 24. Anthropology and Development Studies 25. Education	2003.28–2003.38	8013.12–8013.55
4. Psychology, Psychiatry and Neuroscience	2273.40–2273.44	9093.62–9093.77
34. Art and Design: History, Practice and Theory 35. Music, Drama, Dance and Performing Arts	2439.09–2439.13	9756.38–9756.51
16. Architecture, Built Environment and Planning 26. Sport and Exercise Sciences, Leisure and Tourism	2604.34–2604.35	10417.36–10417.41
17. Geography, Environmental Studies and Archaeology	2831.70–2831.72	11326.81–11326.88
1. Clinical Medicine 2. Public Health, Health Services and Primary Care 3. Allied Health Professions, Dentistry, Nursing and Pharmacy 5. Biological Sciences 6. Agriculture, Veterinary and Food Science	3280.95–3281.02	13123.79–13124.06
15. General engineering 12. Aeronautical, Mechanical, Chemical and Manufacturing Engineering 7. Earth Systems and Environmental Sciences 8. Chemistry 9. Physics 10. Mathematical Sciences 11. Computer Science and Informatics 13. Electrical and Electronic Engineering, Metallurgy and Materials 14. Civil and Construction Engineering	3659.81–3659.88	14639.23–14639.52

The above figures, however, should not be directly used to calculate the total number of outputs submitted by a specific HEI under each UoA because the number of outputs submitted is weighted by the number of people who submitted. Additionally, the monetary value of outputs in different disciplines should not undermine or exaggerate the value of research in different disciplines. Further calculations can be found in the web-appendix MS Excel spreadsheets.

### Does publication of research in high impact journals make it good research?

This section discusses how the research question no. 3 was investigated through examining the relationship between the chosen HEIs percentage of outputs in Q1 journals and percentage of 4* publications. Table 2 indicates the submission characteristics of the five chosen UoAs. HEFCE advised the HEIs that the evaluation is primarily based on ‘originality, significance and rigour’ of the output. However, in its entirety, the evaluation framework becomes a subjective decision of the evaluator. A potential method to recognise quality of an output is to observe the quality or rank of its journal based on the journal impact factor.

**Table 2:** Journal article and 4* statistics of five UoAs submitted for the REF 2014 (REF Executive Summaries).

UoA (HEIs submitted)	Total journal articles submitted (Average per HEI)	Total Q1 outputs (Average per HEI)	Average Q1%	Average 4*% profile of outputs
Clinical Medicine (24)	10986 (457.75)	10452 (435.5)	95.28	23.1
Physics (32)	5302 (165.68)	4762 (148.81)	89.68	21.3
Psy, Psych & Neuro (65)	7484 (115.13)	5560 (85.53)	59.15	25.9
Anthro & Dev Science (20)	1198 (59.9)	400 (20)	30.2	19.1
Classics (18)	345 (19.1)	6 (.33)	1.9	29.4

All the outputs submitted by multiple UK HEIs in the chosen UoAs were filtered for journal article submissions and the impact rating of every article’s journal was mapped using Thomson Reuters’ Journal Citation Reports. All the quartile 1 (Q1) articles were filtered, which allowed the estimation of percentage of Q1 publications in all HEIs in the five UoAs (Tables 3–7).

**Table 3:** HEI’s Q1% and 4*% in clinical medicine UoA.

HEI	4*%	Q1%
The University of Birmingham	17	96.12
University of Bristol	13.5	87.77
University of Cambridge	39.4	96.91
The University of East Anglia	34.5	100
University of Exeter	35.6	95.56
Imperial College London	26.9	98.32
The Institute of Cancer Research	29.2	96.98
King's College London	31.7	98.6
The University of Leeds	18.4	99.42
The University of Leicester	22	93.01
The University of Liverpool	16.4	93.54
Liverpool School of Tropical Medicine	16.4	96.36
University College London	16.7	92.4
London School of Hygiene and Tropical Medicine	22.2	87.96
The University of Manchester	22.3	95.04
University of Newcastle Upon Tyne	18.3	96.09
The University of Nottingham	18.2	94.76
University of Oxford	33.5	96.56
University of Plymouth	48.3	98.33
Queen Mary University of London	26.8	98.09
The University of Sheffield	20.8	93.31
The University of Southampton	16.9	88.21
St. George's, University of London	23.1	95.63
The University of Warwick	24	97.67

**Table 4:** HEI’s Q1% and 4*% in psychology/psychiatry/neuroscience UoA.

HEI	4*%	Q1%
Anglia Ruskin University	10.3	53.85
Birkbeck College	38.5	75.41
The University of Birmingham	36.6	74.51
The University of Bolton	4.2	29.17
Bournemouth University	10.8	62.16
University of Bristol	24	81.37
Brunel University London	8	45.98
University of Cambridge	43.8	93.65
University of Central Lancashire	5.6	40.28
University of Chester	2	48.98
The University of Chichester	0	36.84
The City University	14	64.49
Coventry University	9.8	24.39
University of Derby	3.8	28.3
University of Durham	16	63.83
The University of East Anglia	25.8	74.19
University of East London	7.8	62.5
Edge Hill University	22.7	50
The University of Essex	40	72.86
University of Exeter	31.4	77.97
Goldsmiths' College	18.3	79.13
University of Greenwich	11.6	48.84
University of Hertfordshire	2.2	52.17
The University of Hull	11.2	44.94
Imperial College London	33.2	96.89
The University of Keele	6.1	46.94
The University of Kent	20	71.3
King's College London	24.8	88.4
Kingston University	6.1	45.45
The University of Lancaster	27.7	81.54
The University of Leeds	16	72.92
Leeds Beckett University	2.4	33.33
The University of Leicester	31.4	79.56
University of Lincoln	7.1	38.1
The University of Liverpool	23.1	74.07
Liverpool Hope University	2.4	30.95
Liverpool John Moores University	16.1	48.39
University College London	33.3	83.24
London South Bank University	5.7	51.43
The University of Manchester	23.6	78.55
Middlesex University	6.5	63.04
University of Newcastle Upon Tyne	28.4	81.22
Newman University	0	36.36
The University of Northampton	0	14.81
University of Northumbria at Newcastle	10.9	53.13
The University of Nottingham	22.8	69.86
Nottingham Trent University	16.4	52.05
University of Oxford	54	93.93
Oxford Brookes University	12.2	39.02
University of Plymouth	19.3	62.28
University of Portsmouth	16	52
The University of Reading	18.6	70.51
Roehampton University	10.4	39.58
Royal Holloway, University of London	41.5	74.39
The University of Sheffield	22	84.75
The University of Southampton	28.3	72.57
Staffordshire University	2.4	21.43
The University of Surrey	21.2	69.7
University of Sussex	35.4	88.19
The University of Warwick	40.5	73.81
The University of Westminster	10.2	63.27
University of Winchester	2.8	33.33
University of Worcester	3.7	11.11
The University of York	43.4	83.13
York St John University	7.1	21.43

**Table 5:** HEI’s Q1% and 4*% in Physics UoA.

HEI	4*%	Q1%
The University of Bath	15.5	90.48
The University of Birmingham	22.9	92.95
University of Bristol	18.8	78.01
University of Cambridge	23.9	92.7
University of Central Lancashire	9.5	88.1
University of Durham	21.8	93.84
University of Exeter	21.9	95.77
University of Hertfordshire	8.5	91.54
The University of Huddersfield	9.5	47.62
Imperial College London	23.6	88.44
The University of Keele	23.3	100
The University of Kent	23.5	100
King's College London	22.7	92.71
The University of Lancaster	27.6	87.31
The University of Leeds	13.6	95.45
The University of Leicester	9	79.4
The University of Liverpool	17.4	81.88
Liverpool John Moores University	22.4	100
University College London	18.6	89.64
Loughborough University	6.7	82.67
The University of Manchester	17.6	83.86
The University of Nottingham	20.7	97.93
University of Oxford	33.2	90.75
University of Portsmouth	21.6	100
Queen Mary University of London	23.1	94.51
Royal Holloway, University of London	17.8	82.65
The University of Sheffield	23.6	94.55
The University of Southampton	25	93.33
The University of Surrey	15.8	89.11
University of Sussex	20	96.84
The University of Warwick	24.1	95.35
The University of York	18.2	82.48

**Table 6:** HEI’s Q1% and 4*% in anthropology & development studies UoA.

HEI	4*%	Q1%
Brunel University London	14.7	8.7
University of Cambridge	22	36.14
University of Durham	27.4	44.12
The University of East Anglia	23	37.78
Goldsmiths' College	17.9	13.64
University of Greenwich	0	20.83
The University of Kent	8.7	57.14
Liverpool John Moores University	16.1	53.33
University College London	26.3	41.27
The London School of Economics and Political Science	31.5	20
The London School of Economics and Political Science	20.8	30.65
The University of Manchester	27.3	32.95
The University of Manchester	21.4	15.63
The Open University	12.5	21.21
The School of Oriental and African Studies	23.9	16.67
The School of Oriental and African Studies	15.3	32.95
University of Oxford	16.8	36.89
University of Oxford	23.3	29.57
Roehampton University	16.3	34.48
University of Sussex	13.4	20

**Table 7:** HEI’s Q1% and 4*% in classics UoA.

HEI	4*%	Q1%
The University of Birmingham	32.9	20
University of Bristol	32	0
University of Cambridge	38.8	0
University of Durham	35.3	0
University of Exeter	22.1	0
The University of Kent	16.7	0
King's College London	33.9	6.45
The University of Leeds	19	0
The University of Liverpool	21.2	0
University College London	19.5	0
The University of Manchester	21.7	0
University of Newcastle Upon Tyne	31.6	0
The University of Nottingham	38.9	6.25
The Open University	10	0
University of Oxford	34.3	1.49
The University of Reading	37.3	0
Royal Holloway, University of London	18.2	0
The University of Warwick	26.4	0

**Fig 1:**
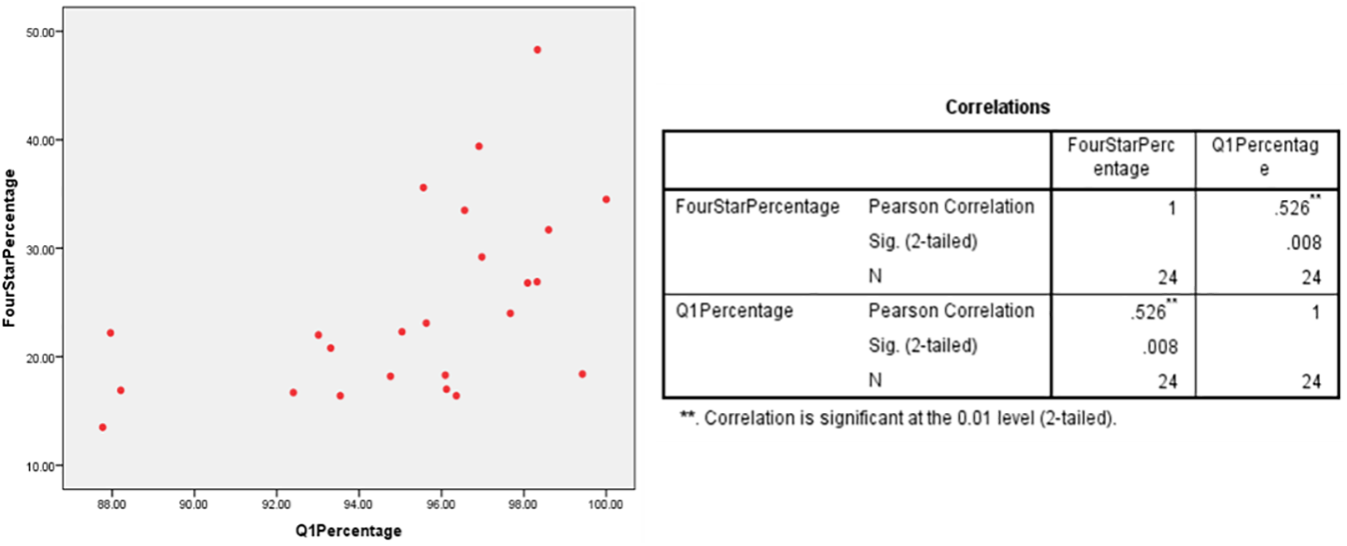
4*% vs Q1% of various HEIs in clinical medicine UoA.

**Fig 2:**
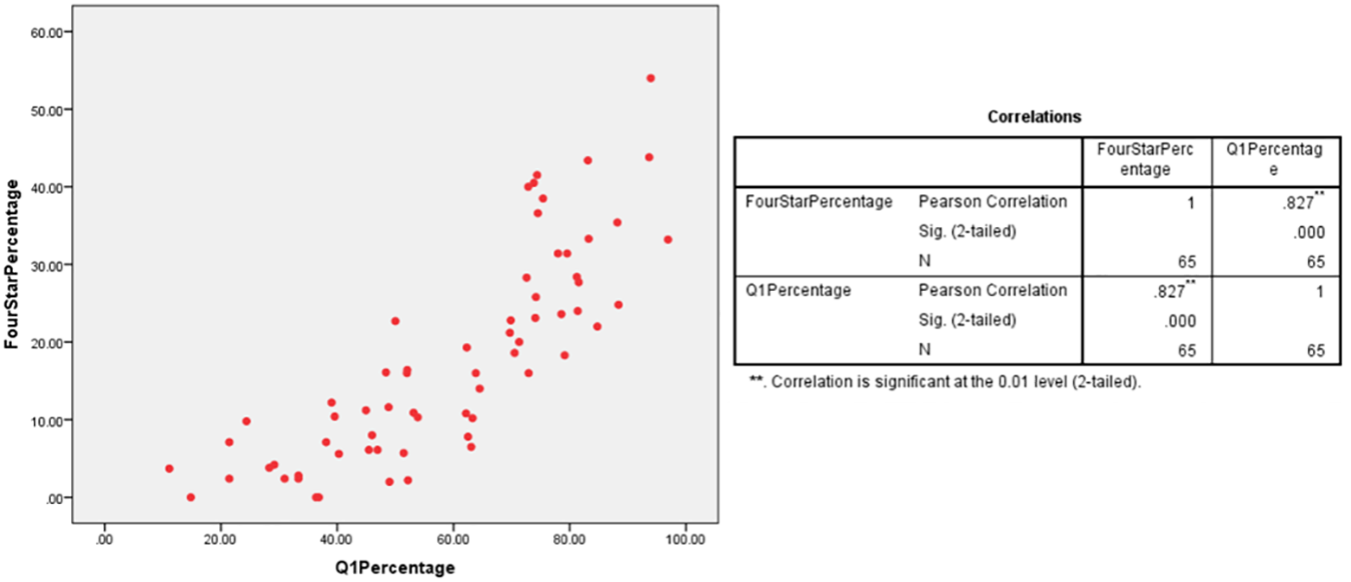
4*% vs Q1% of various HEIs in psychology/psychiatry/neuroscience UoA.

**Fig 3:**
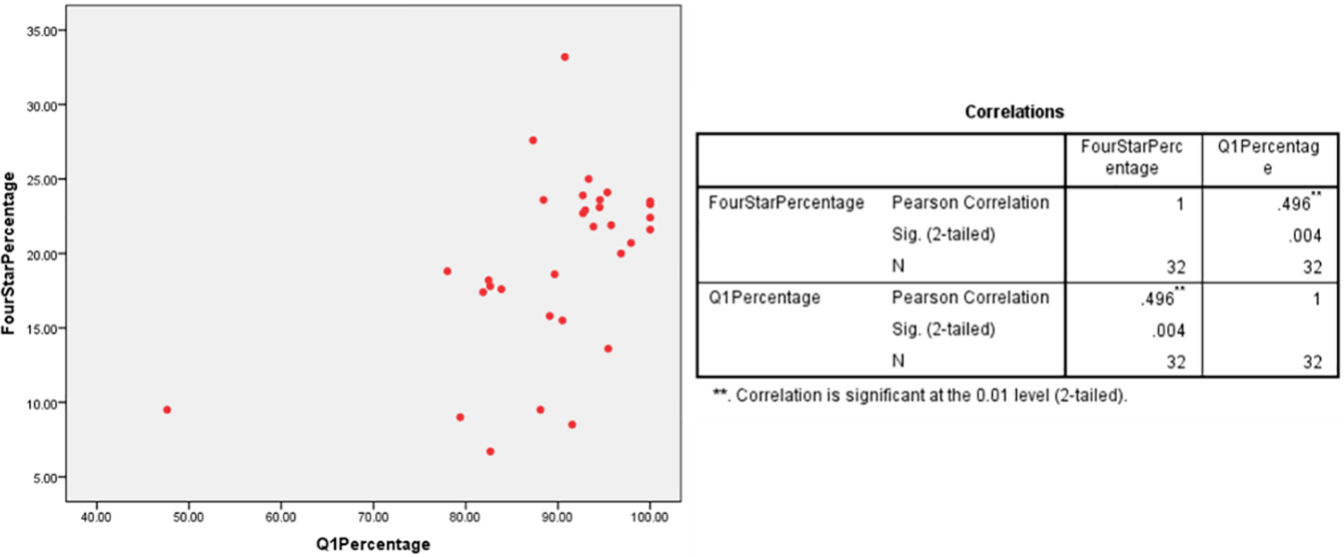
4*% vs Q1% of various HEIs in physics UoA.

**Fig 4:**
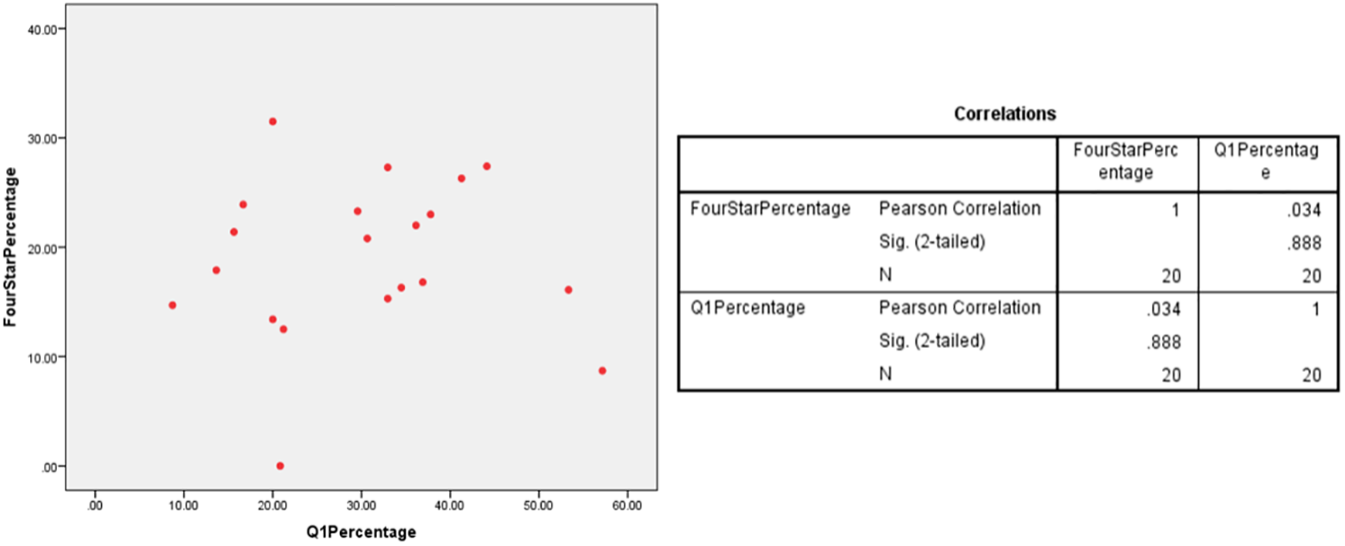
4*% vs Q1% of various HEIs in anthropology/development studies UoA.

**Fig 5:**
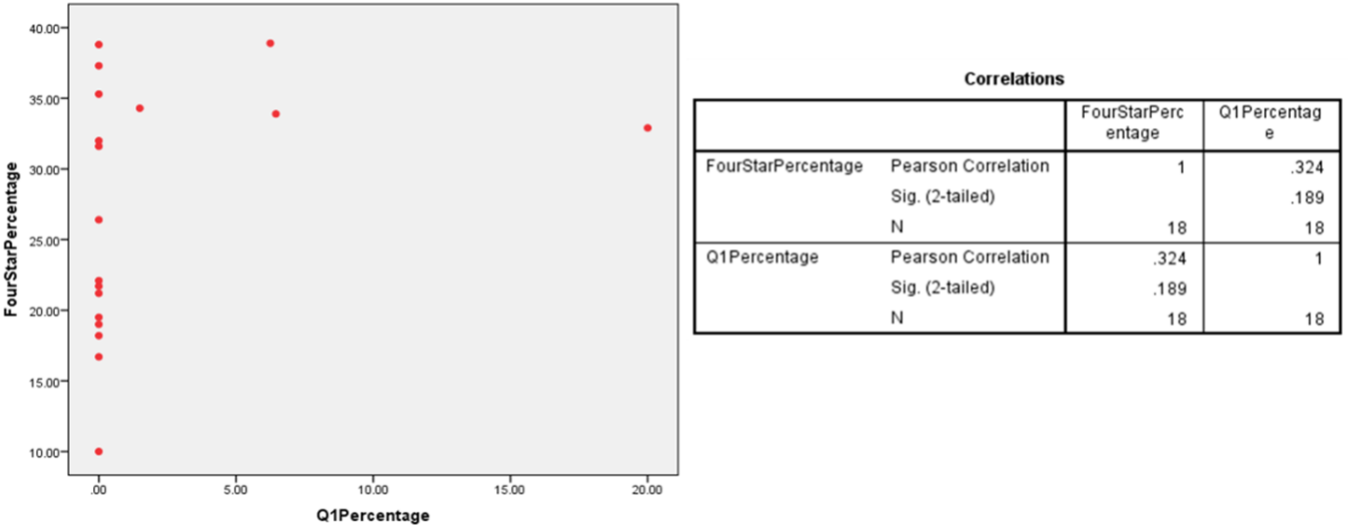
4*% vs Q1% of various HEIs in classics UoA.

A scatter plot was employed to observe any linear relationship between percentage of 4* outputs and percentage of Q1 publications in multiple UK HEIs which had submitted under the five UoAs. The different plots indicate a linear relationship between the percentages of Q1 publications and 4* outputs at various HEIs in the Clinical Medicine (r = 0.526/ n = 24/ p = 0.008), Physics (r = 0.496/ n = 32/ p = 0.004) and Psychology/Psychiatry/Neuroscience (r = 0.827/ n = 65/ p = 0) UoAs (Figs 1–3). However, no relationship was found for the Classics (r = 0.324/ n = 18/ p = 0.189) and Anthropology/Development Studies (r = 0.034/ n = 20/ p = 0.888) UoAs (Figs 4 and 5).

Exploring further, we performed a simple linear regression for UoAs 1, 4 and 9 to investigate if Q1 percentage is a good predictor of 4* percentage a HEI can achieve.

For UoA 1 (Clinical medicine), the ANOVA indicated model significance (F[1, 22] = 8.417, p = .008) and 24.4% of variance in 4* percentage can be explained by a HEI’s Q1 percentage. The line equation to predict 4* percentage is *y = 1*.*332(x)+(-102*.*245)* which is significant (t = 2.901, p = .008).

For UoA 4 (Psychology/Psychiatry/Neuroscience), the ANOVA indicated model significance (F[1, 63] = 135.977, p<0) and 67.8% of variance in 4* percentage can be explained by a HEI’s Q1 percentage. The line equation to predict 4* percentage is *y = 0*.*514(x)+(-12*.*614)* which is significant (t = 11.661, p<0).

For UoA 9 (Physics), the ANOVA indicated model significance (F[1, 30] = 9.772, p = .004) and 22.1% of variance in 4* percentage can be explained by a HEI’s Q1 percentage. The line equation to predict 4* percentage is *y = 0*.*302(x)+(-7*.*648)* which is significant (t = 3.126, p = .004). For UoA 9, this test was important as an outlier could have skewed the dataset, however as Fig 3 indicates, the relationship is linear at the further end of the x and y axis.

UoA 4 appears to have the strongest link with an r value (Pearson correlation) of 0.827, although the r values are not exceptionally high in clinical medicine and physics, the scatter plot explains the trend of Q1 publications scoring high in the REF. The findings indicate that the outcome of judgements made on the quality of research either by peer-reviewed government ranking (REF results) and metrics-based ranking (JIF) largely remain the same in disciplines where journals are considered the main channels of research communication. There is ample literature suggesting the relationship between expert review decisions and bibliometrics [18–21]. A similar study on Italy’s national research assessment exercise found similar claims that in pure and natural sciences, the perspectives on quality of research is either similar or superior to national research assessment exercises [22]. This study supports this claim, implying that quantitative measures are capable of evaluating research quality that are comparable to expert review based government research assessment [22]. Additionally, such a system instils public trust in the utilisation of public funds in HEIs as performance metrics are readily available for public view [23]. However, JIF has also been indicated to inefficiently evaluate the quality of research and quality mercantilism in general isn’t an appropriate evaluation technique [20, 24–26].

### Can the value of a good research output inform an HEI’s policies?

This section discusses how the research question no. 4 was investigated. The REF’s executive summaries supplied complete information about various UoA’s output submissions, HEIs submitted, category A staff, early career researchers, average 4* and 3* percentages. This assisted in calculating average submissions of each HEI, number of outputs rated 4* and 3*, average submissions per staff number and average number of submissions submitted per staff number rated as 4* and 3*. Category C staff’s outputs were used solely to rate, however were not considered for funding, thus were excluded from our analysis. The average 4* and 3* submissions per staff member as mentioned in the last two columns of Table 8 inform their potential contribution of performance-based funding to the HEI in the UK. For example, a single staff member in the Area Studies UoA submitted 3.58 outputs out of which 0.84 and 1.42 outputs are rated 4* and 3* respectively. Taking these average figures it is possible to predict the income generated by an average member of staff through their REF outputs. Considering a hypothetical situation where an HEI’s department has 5 staff members, they can produce 4.2 outputs of 4* quality and 7.1 outputs of 3* quality out of the 17.9 outputs they would have submitted. The value of 4* and 3* outputs in Area Studies UoA is £7505 and £1876 respectively, which when multiplied by the number produced 4* and 3* outputs and summated gives the total funding the staff have contributed to the HEI, which in this case is £44840.6.

**Table 8:** Average submission characteristics of various UoAs.

Units of Assessment	Av submissions/ HEI	% of 4*	% of 3*	Av no of 4*	Av no of 3*	Av CatA staff submitted	Av submision per staff	4*: CatA Staff	3*: CatA Staff
Area Studies	75.08	23.6	39.7	17.72	29.81	21	3.58	0.84	1.42
Modern Languages and Linguistics	86.71	24.8	42.3	21.50	36.68	23.78	3.65	0.90	1.54
English Language and Literature	77.89	28.6	41.7	22.28	32.48	22.14	3.52	1.01	1.47
History	77.8	27.8	42.7	21.63	33.22	21.51	3.62	1.01	1.54
Classics	63.09	29.4	41	18.55	25.87	17.4	3.63	1.07	1.49
Philosophy	54.35	26.3	42.8	14.29	23.26	14.77	3.68	0.97	1.57
Theology and Religious Studies	47.33	23.7	38.4	11.22	18.17	12.51	3.78	0.90	1.45
Communication, Cultural and Media Studies/Library and Information Management	52.55	23.4	39.3	12.30	20.65	13.95	3.77	0.88	1.48
Economics and Econometrics	92.85	27.7	48.9	25.72	45.40	27	3.44	0.95	1.68
Business and Management Studies	120.83	20.5	42.8	24.77	51.72	32.87	3.68	0.75	1.57
Law	82.46	20.1	47.1	16.57	38.84	23.17	3.56	0.72	1.68
Politics and International Studies	77.98	20.9	40.1	16.30	31.27	22.76	3.43	0.72	1.37
Social Work and Social Policy	77.16	19.4	44.3	14.97	34.18	21	3.67	0.71	1.63
Sociology	90.68	19.7	47.9	17.86	43.44	24.27	3.74	0.74	1.79
Anthropology and Development Studies	80.6	19.1	39.3	15.39	31.68	22.48	3.59	0.68	1.41
Education	72.71	21.7	39.9	15.78	29.01	18.97	3.83	0.83	1.53
Psychology, Psychiatry and Neuroscience	111.29	25.9	45.8	28.82	50.97	30.73	3.62	0.94	1.66
Art and Design: History, Practice and Theory	75.66	18.5	42.6	14.00	32.23	19.09	3.96	0.73	1.69
Music, Drama, Dance and Performing Arts	50.72	25	37.1	12.68	18.82	13.59	3.73	0.93	1.38
Architecture, Built Environment and Planning	84.02	22.7	40.7	19.07	34.20	22.77	3.69	0.84	1.50
Sport and Exercise Sciences, Leisure and Tourism	54.09	19.5	42.2	10.55	22.83	15.49	3.49	0.68	1.47
Geography, Environmental Studies and Archaeology	81.36	22.1	42.1	17.98	34.25	22.78	3.57	0.79	1.50
Clinical Medicine	432.41	23.1	53.5	99.89	231.34	115.19	3.75	0.87	2.01
Public Health, Health Services and Primary Care	152.53	22.6	48.6	34.47	74.13	42.31	3.61	0.81	1.75
Allied Health Professions, Dentistry, Nursing and Pharmacy	110.19	21.4	55.7	23.58	61.38	29.23	3.77	0.81	2.10
Biological Sciences	195.63	29.3	48.9	57.32	95.66	53.93	3.63	1.06	1.77
Agriculture, Veterinary and Food Science	135.17	18.2	50.7	24.60	68.53	35.93	3.76	0.68	1.91
General engineering	140.27	17.2	65.8	24.13	92.30	39.46	3.55	0.61	2.34
Aeronautical, Mechanical, Chemical and Manufacturing Engineering	166.16	18	60.4	29.91	100.36	46.08	3.61	0.65	2.18
Earth Systems and Environmental Sciences	116.66	18.2	60.7	21.23	70.81	30.66	3.80	0.69	2.31
Chemistry	126.97	22.1	69.4	28.06	88.12	33.21	3.82	0.84	2.65
Physics	157.21	21.3	66.6	33.49	104.70	41.56	3.78	0.81	2.52
Mathematical Sciences	131.98	22.7	59.7	29.96	78.79	36.41	3.62	0.82	2.16
Computer Science and Informatics	86.12	22.1	47.1	19.03	40.56	22.96	3.75	0.83	1.77
Electrical and Electronic Engineering, Metallurgy and Materials	108.86	19.7	67.7	21.45	73.70	28.94	3.76	0.74	2.55
Civil and Construction Engineering	98.85	18.1	58	17.89	57.33	27.85	3.55	0.64	2.06

As the results are based on averages, HEIs can set themselves benchmarks to improve their performance through internal evaluations and predicting their performance in the future research assessment exercises becomes a possibility. The results inform an HEI by allowing it to take strategic decisions through altering its policies in the following ways:

1. It informs an HEI the amount of funding an academic can bring into the department.
2. Predict the future income for a given department based on the number of staff.
3. Interdisciplinary research sits in two different departments. For example, information sciences either come under UoA 11 or UoA 36. As UoA 11 offers higher income for good research outputs, HEIs which have submitted their information sciences research in UoA 36 may consider submitting in UoA 11 for the next exercise.
4. The results assist HEIs investment and financial strategy by informing their potential income generation through performance-based research funding. For example, an HEI can recruit more academics in the Engineering department so as to increase their chances of acquiring funding. Hence, the investment decision can influence the future of science.

## Conclusion

Our investigation of the REF as a case study reveals that in the UK a world leading research output earns £7504 to £14,639 and an internationally excellent research output earns £1876 to £3659, varying according to their areas of research, per year in a REF cycle. This answers our inquiry into knowing the monetary value of a good research output and subsequent disciplinary differences. Although this assigned monetary value of research output is dependent on a country’s budget, it has implications for the progress of science and research. For example,

- The results can provide a reference to compare the monetary value of good research outputs in different countries i.e. Italy’s Research Quality Evaluation (VQR), Netherlands’s Standard Evaluation Protocols (SEP). According to HESA (2013), the funding pot available for UK universities significantly reduced from 2008, which was recently addressed by the Universities UK’s 2015 call to increase science research funding [27].
- The figures obtained through this investigation would allow the HEIs to forecast and build strategies for investment in different disciplines that may have implications for the progress of science and research in general.
- Additionally, this investigation can be applied by UK HEIs into their strategies of submission for the next research assessment exercise. This answers our inquiry to know the potential policy implications arising by extricating the monetary value of good research outputs.

Our further investigation to observe any relationship between reputation of publication source and quality of a research output revealed a linear relationship between the percentage of quartile-one (Q1) journal publications and funding allocation in the Clinical Medicine, Physics and Psychology/Psychiatry/Neuroscience UoAs, and no relationship was found in the Classics and Anthropology/Development Studies UoAs, due to the fact that most publications in the latter two disciplines are not journal articles. This partly answers our final question and therefore we recommend a similar investigation into the rest of the thirty-one UoAs which would offer a clearer picture, adding, the existence of academic literature either confirming the relationship or refuting it [25, 28, 29].

## Supporting information

S1 File FTE RoI Outputs. (XLSX)

S2 File Value of a research output in various UoAs. (XLSX)
