# Correction: Metric-based vs peer-reviewed evaluation of a research output: Lesson learnt from UK’s national research assessment exercise

**DOI:** 10.1371/journal.pone.0190337

**Published:** 2017-12-20

**Authors:** Kushwanth Koya, Gobinda Chowdhury

In the Methods section, there is an error in the fourth equation in the subsection titled “‘Example of the calculation”. Please view the complete, correct equation here:

(265.09*14639.38)+(395.51*3659.84)=£5328256.5[£5328295approx.]
